# Lamb-Wave-Based Multistage Damage Detection Method Using an Active PZT Sensor Network for Large Structures

**DOI:** 10.3390/s19092010

**Published:** 2019-04-29

**Authors:** M. Saqib Hameed, Zheng Li, Jianlin Chen, Jiahong Qi

**Affiliations:** State Key Laboratory for Turbulence and Complex Systems, Department of Mechanics and Engineering Science, College of Engineering, Peking University, Beijing 100871, China; saqib@pku.edu.cn (M.S.H.); cjl@pku.edu.cn (J.C.); 1401214512@pku.edu.cn (J.Q.)

**Keywords:** Lamb wave, PZT transducer, multistage damage quantification, Gabor wavelet transform, elliptical reconstruction

## Abstract

A multistage damage detection method is introduced in this work that uses piezoelectric lead zirconate titanate (PZT) transducers to excite/sense the Lamb wave signals. A continuous wavelet transformation (CWT), based on the Gabor wavelet, is applied to accurately process the complicated wave signals caused by the damage. For a network of transducers, the damage can be detected in one detection cell based on the signals scattered by the damage, and then it can be quantitatively estimated by three detection stages using the outer tangent circle and least-squares methods. First, a single-stage damage detection method is carried out by exciting a transducer at the center of the detection cell to locate the damaged subcell. Then, the corner transducers are excited in the second and third stages of detection to improve the damage detection, especially the size estimation. The method does not require any baseline signal, and it only utilizes the same arrangement of transducers and the same data processing technique in all stages. The results from previous detection stages contribute to the improvement of damage detection in the subsequent stages. Both numerical simulation and experimental evaluation were used to verify that the method can accurately quantify the damage location and size. It was also found that the size of the detection cell plays a vital role in the accuracy of the results in this Lamb-wave-based multistage damage detection method.

## 1. Introduction

Among structural health monitoring (SHM) techniques, Lamb-wave-based methods have shown great potential for fast damage detection in plate-like structures [1]. Lamb waves can propagate a distance with low attenuation and are highly sensitive to small imperfections [2,3]. These waves have been significantly utilized for SHM and determination of surface defects in large metallic structures [4]. Accurately estimating the damage not only requires the construction of damage images for its localization but also an accurate quantification of both the location and size of the damage. The presence of damage in a structure results in the forward and backward scattering of waves. The change in Lamb wave propagation due to this reflection and transmission can be detected and analyzed to evaluate the damage [5]. There are a variety of methods to excite and receive Lamb wave signals. These methods can be grouped into five broad categories based on the transducers [6]: ultrasonic probes, lead zirconate titanate (PZT) transducers and piezocomposite transducers, laser-based ultrasonic transducers, interdigital transducers (IDTs), and fiber-optic transducers. Comparing these methods, PZT transducers can be directly mounted on the surface of the target structure to excite/receive the Lamb waves through in-plane strain coupling [7,8]. As a result, PZT transducers can be used for fast and in situ SHM. PZT transducers are small in size, light weight, consume a low amount of power, and produce a frequency response in a wide region. They can be conveniently arranged as a network of sensors to record multipoint measurements under and on the surface of the host structure. One prominent feature of PZT transducers for damage detection is that their performance does not deteriorate even when the damage occurs in its vicinity [6,9]. Lamb-wave-based damage detection techniques have employed different arrangements of PZT transducers to actuate and sense the wave propagation. The rectangular/square array of sensors [10,11,12] and circular arrangement of sensors [11,13,14,15,16] are popular strategies to excite/sense Lamb waves in homogeneous plates. The circular arrangement [13] was further improved using two ring-shaped arrangements of PZT transducers on an aluminum plate [17]. This pitch–catch configuration reduced the isolated region and improved the resolution in the damage monitoring area [17]. Also, four PZT discs have been arranged on the wing slat of Boeing 737 aircraft [18], and three PZT sensors have been attached on the surface of a composite plate [19] to excite and sense the Lamb waves.

Lamb waves generated by PZT transducers contain dispersion and multimode characteristics and require sophisticated signal-processing techniques to process the dynamic response signals [6]. Various approaches have been introduced for physical interpretation of the response signal and damage estimation in target structures. Discrete wavelets and the Hilbert transform have been combined to process Lamb wave signals [13], and to estimate the damage location and size using arrival time and amplitude of the damage-scattered peak [18]. Empirical mode decomposition (EMD) has been used to decompose the scattered signal in a damaged plate to obtain the damage size and location [19]. Wavelet transforms have been used in dispersion analysis of Lamb waves and detection of damage in aluminum plates [20]. An enhanced detection resolution usually requires more measurement points and the processing of a large quantity of data, which makes the detection time consuming. Damage detection based on Lamb wave focusing by an array of PZT transducers has improved the detection resolution using dispersion compensation [2]. An approach for estimating guided wave scattering patterns from a sparse transducer array was presented by recording the corresponding scattering matrix at a finite number of incident and scattered angles. The recorded samples were further used to estimate the scattering matrix by a multiquadric radial basis function (MQ-RBF) interpolation method [21]. An ellipse-based damage-imaging algorithm was developed to investigate linear cracks in a steel plate using guided waves [22]. It was found that only limited size defects could be mapped, and defect location or size could not be effectively quantified. Lamb-wave-based crack quantification for plate-like structures has been developed by finite element (FE) simulation and Bayesian updating of data [23]. It was found that improving the accuracy required using more updating data points. There have also been methods which dealt with signal processing to improve the damage localization in the existing detection techniques. A multifrequency analysis [24] used the time shift averaging algorithms applied to the differential signals filtered at multiple frequencies to detect the hole damage in an aluminum plate. It resulted in many images obtained for the same structural state which were then fused to improve the damage localization. A multiparameter approach [25] was presented using the reconstruction algorithm which enhanced the damage localization of single detection framework by extracting different features from the propagating wave. Furthermore, there have been Lamb-wave-based baseline-free methods for damage imaging and localization in isotropic plates using wavelet transform, empirical mode decomposition [26], and instantaneous baseline measurement [27] methods. The instantaneous baseline damage localization for stiffened composite panels using the delay-and-sum algorithm [28] has also been studied. 

The methods utilizing PZT transducers usually require a large number of data points and the baseline signal for comparison with the damaged signal for accurate damage estimation. The total focusing method (TFM) used 16 PZT transducers arranged on an aluminum plate and required 240 signals per assessment to detect the circular damage. It further required the baseline signal in order to determine the initial conditions such as wave speed [14]. The reconstruction algorithm for the probabilistic inspection of damage (RAPID) technique based on the ellipse method used 16 PZT transducers to calculate the area of circle-like damage [11]. A two-step method that simulated corrosion as circular damage in an aluminum plate required 6 PZT transducers as well as the baseline signal for damage imaging [15]. A data-driven, matched-field processing technique to locate circular damage required 17 PZT transducers randomly distributed on an aluminum plate. A strategy used the combination electromechanical impedance–PZT (EMI–PZT)-based technique and the convolutional neural network (CNN) algorithm to detect a 12-mm-diameter metallic nut (simulated as circular damage) in an aluminum plate [12]. An ellipse algorithm model based on Voronoi tessellation and Delaunay triangulation used 60 transducers for the best coverage of a hole [29]. Similarly, other nondestructive testing (NDT) and SHM methods have used 8 [13], 12 [16], and 16 [14] PZT transducers for an accurate damage estimation. However, the use of too many PZT transducers or comparing the damaged signal with the baseline requires a great amount of data to be processed during the NDT/SHM process.

There are few Lamb-wave-based methods that detect damage using two or more stages/levels to improve the detection accuracy. A two-stage algorithm was proposed for an aluminum plate [10] to locate the damage using signals from undamaged and damaged plates. Damage location was estimated in the first stage by applying the CWT on the extracted signals, and the results further improved in the second stage using Euclidean and Lagrangian optimization techniques on an enclosed area. This imaging method requires knowledge of the baseline signal, does not quantify the damage location, and provides no estimation of the damage size. A three-level damage detection approach was presented [30] which used different methods at different levels to improve the damage localization. Level 1 is based on finding the maximum energy difference in damaged and undamaged structures. Level 2 measured the EMI, and level 3 characterized the damage by utilizing a weighted energy arrival method (WEAM). A multiple damage detection approach has been introduced using different stages of detection, and the influence of variable velocity on the damage estimation was analyzed [31]. An NDT technique for Lamb wave tomography (LWT) was utilized [32] for two-stage reconstruction of damage images. Two sets of parallel scans were used to approximate damage location and size in the first stage. In the second stage, small damage-related parameters, such as the changes in wave attenuation coefficient and wave velocity, were computed through linear approximations. Another two-step method for damage localization on an aluminum plate is to detect damage in the first step and then perform localization and characterization in the second step using a delay-and-sum imaging method [15]. A Lamb-wave-based sparse representation inspection strategy has been proposed [33] which uses the l1-norm optimization algorithm. The severity of damage was estimated by Euler–Bernoulli beam theory based on using baseline and damaged signals. The method introduced two damage detection levels: damage identification and depth evaluation. Two Lamb-wave-based predictive circle methods for damage estimation were compared [34]. The first method used six intersection points of the four elliptical wave paths to estimate the damage represented by an enclosed circle. The second method drew a circle tangent to these ellipses, which represented the damage better than the first method. However, the accuracy of the methods was tested in two different sizes of the inspection area, and signals were processed using the cross-correlation technique, which requires the baseline signal. By now, the accuracy of results in two-stage damage detection methods has improved in the second stage, usually by using additional data points or introducing a new signal-processing technique. In some methods, the feature measured in the second stage is different from the one measured in the first stage (e.g., location of the defect in the first stage and its size in the second stage). Most of the existing NDT methods for damage detection in plate-like structures only focus on identification of the damage position and not its extent/size [10,14,15,22,24,25,30,31,32,33,35,36,37]. The reason is that most of the existing methods detect the damage usually in the form of images, and do not provide accurate quantitative measurements. 

In order to provide a convenient multistage damage detection method in practice to meet the different requirements for detection accuracy, a Lamb-wave-based multistage damage detection method is introduced in this research to accurately quantify not only the location but also the size of the damage. The method introduces a strategy to arrange PZT transducers in the form of a network of detection cells on a large plate-like structure. The CWT method using the Gabor wavelet is applied to analyze the dynamic response caused by the damage. The damage location and size are evaluated in three stages through an algorithm based on the outer tangent circle method. A single-stage method provides an initial estimate of the damage size and locates the damaged cell. It further locates the damaged subcell inside the damaged cell. The estimation of damage location and size inside the damaged subcell significantly improves in the second and third stages of detection. The arrangement of transducers and the signal-processing method remain the same in all stages of damage detection. The method does not require the baseline signal from the undamaged plate, and the improvements in the subsequent stages can be achieved without the need for additional transducers. These features make the method less complicated, low-cost, and less time consuming.

The organization of this article is as follows: Section 2 introduces the theoretical background of the outer tangent circle method. The detection strategy of the single-stage method, including the dispersion curves, FE simulation model, and influence of the size of the detection cell, are presented in Section 3. The multistage stage damage detection in Section 4 includes two- and three-stage detection strategies. Section 5 covers the application of the method for multiple damage cases. The verification of the method through experiments is presented in Section 6. At the end, the article is summed up with a discussion of the results in Section 7, and conclusive remarks in Section 8. 

## 2. Outer Tangent Circle Method

**Figure 1:**
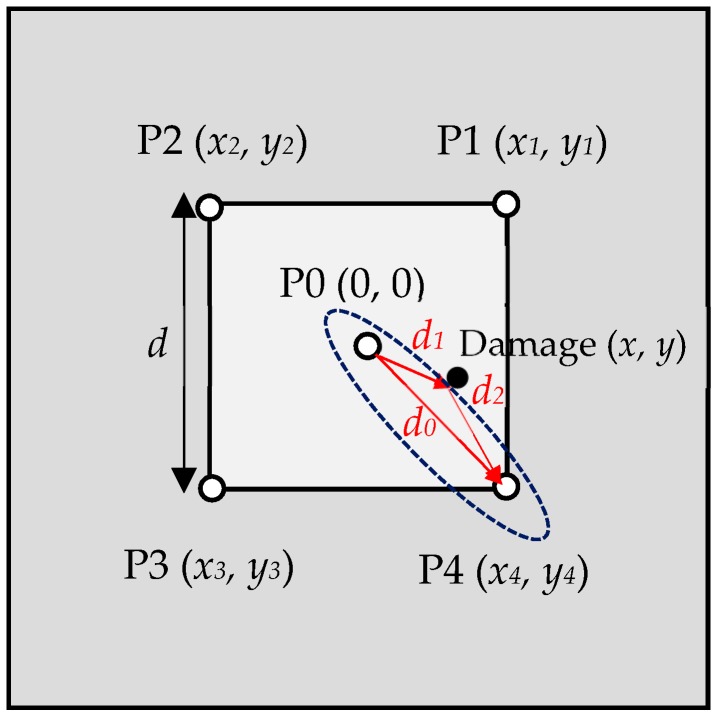
Damaged plate with lead zirconate titanate (PZT) transducer configuration and an ellipse tangent to the damage.

Consider a plate with the central PZT transducer (P0) acting as an actuator, as shown in Figure 1, to induce waves that propagate radially outward towards the free boundaries of the plate. Imagine circular damage located at coordinates (*x*, *y*) from the excitation point. Four other PZT transducers (P1–P4 in Figure 1) act as sensors and are arranged in a square configuration beyond the damage location. The four corner transducers receive the direct signal from P0 and also the signals scattered by the damage. For example, the direct signal from P0 propagates a distance $d_{0}$ and arrives at P4 with a time of flight $t_{040}$. However, there is another signal received at P4 by the scattering from the damage that travels a longer distance $d_{1}+\;d_{2}$ and requires a longer arrival time $t_{041}+t_{042}$. The difference in the time of flight between the direct and damage-scattered signals is the delay time $\Delta t_{04}$, as shown in Figure 1, which can be calculated from

(1) $$\frac{d_{1}}{v}+\frac{d_{2}}{v}-\frac{d_{0}}{v}=(t_{041}+t_{042})-t_{040}=\Delta t_{04}$$

where v is the group velocity. The distances $d_{0},\;d_{1},\;\mathrm{and}\;d_{2}$ can be calculated using the geometric relations in Figure 1 by

(2) $$\begin{array}{c} d_{0}=\frac{\sqrt{2}}{2}d,\\ d_{1}=\sqrt{{\left(x-0\right)}^{2}+{\left(y-0\right)}^{2}}=\sqrt{x^{2}+y^{2}}\\ d_{2}=\sqrt{{\left(x_{4}-x\right)}^{2}+{\left(y_{4}-y\right)}^{2}} \end{array}\;,\;\;$$

where *d* is the distance between the corner transducers. Substitution into Equation (1) results in the relation

(3) $$\sqrt{x^{2}+y^{2}}+\sqrt{{\left(x_{4}-x\right)}^{2}+{\left(y_{4}-y\right)}^{2}}-\frac{\sqrt{2}}{2}d=v\cdot \Delta t_{04}.$$

For a known value of $\Delta t_{04}$, Equation (3) represents an ellipse with focal points at P0 and P4, as shown with the blue dashed line in Figure 1. Similarly, if the delay times for P1, P2, and P3 are $\Delta t_{01}$, $\Delta t_{02},$ and $\Delta t_{03}$, respectively, then there will be three more ellipses with focal points at P0–P1, P0–P2, and P0–P3, respectively. Therefore, a total of four ellipses can be drawn by

(4) $$\begin{array}{c} \sqrt{x^{2}+y^{2}}+\sqrt{{\left(x_{1}-x\right)}^{2}+{\left(y_{1}-y\right)}^{2}}=v\cdot \Delta t_{01}+\frac{\sqrt{2}}{2}d\\ \sqrt{x^{2}+y^{2}}+\sqrt{{\left(x_{2}-x\right)}^{2}+{\left(y_{2}-y\right)}^{2}}=v\cdot \Delta t_{02}+\frac{\sqrt{2}}{2}d\\ \sqrt{x^{2}+y^{2}}+\sqrt{{\left(x_{3}-x\right)}^{2}+{\left(y_{3}-y\right)}^{2}}=v\cdot \Delta t_{03}+\frac{\sqrt{2}}{2}d\\ \sqrt{x^{2}+y^{2}}+\sqrt{{\left(x_{4}-x\right)}^{2}+{\left(y_{4}-y\right)}^{2}}=v\cdot \Delta t_{04}+\frac{\sqrt{2}}{2}d. \end{array}$$

Since the Lamb wave is scattered from the outer periphery of the damage, these ellipses will remain tangent to the damage periphery. A circle externally tangent to these ellipses will exactly cover the damage area. An algorithm based on the least-squares method was designed to draw the four ellipses using Equation (4), and to find the center and size of the externally tangent circle. The center of the externally tangent circle gives the location (*x*, *y*) of damage, and the diameter of this circle provides the size.

## 3. Single-Stage Damage Detection Method

### 3.1. Dispersion Curves

**Figure 2:**
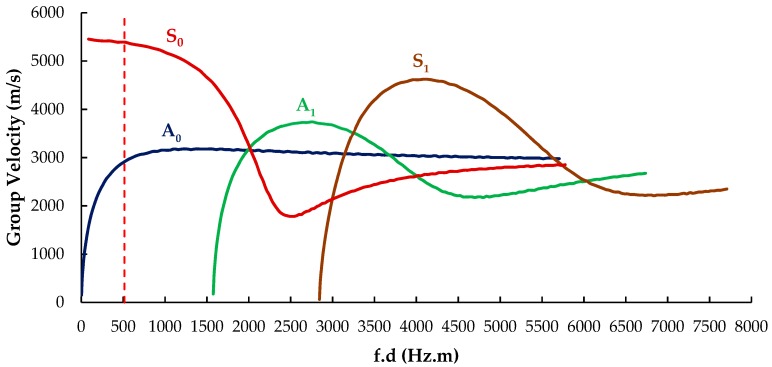
Group velocity dispersion curves for aluminum plate.

An infinite number of guided wave modes can be excited in a thin plate with two surfaces. The phase and group velocities of Lamb waves vary as the product of signal frequency and thickness of the plate varies. The dispersion curves for Lamb waves in a thin aluminum plate were drawn using the reverberation-ray matrix method [38], which shows both symmetric (S) and antisymmetric (A) modes propagating simultaneously, as shown in Figure 2.

In the low-frequency range, only two modes of Lamb waves (S_0_, A_0_) propagate, with the group velocity of the S_0_ mode being significantly higher than the A_0_ mode. If one uses a modified signal with a narrow frequency band, the S_0_ and A_0_ modes can be identified separately with the peak of the S_0_ mode arriving first and the A_0_ mode arriving second. In the mode conversion, after reflection from the damage, S_0_ will reflect a faster symmetric mode S_0_ and a slower antisymmetric mode A_0_ [39]. In the majority of studies, the S_0_ mode is selected for damage detection because it has lower attenuation, fast propagation velocity, and lower dispersion in comparison with the A_0_ mode. Therefore, the scattered S_0_ mode is also easy to identify as the one propagating faster [40,41], and it was exclusively considered for the damage detection method proposed here. 

### 3.2. FE Simulation Model

The numerical simulations were performed on an 800 × 800 × 1.293 mm square aluminum plate using the commercial FE software ABAQUS explicit. The material properties used were E=71 GPa, υ = 0.3, and ρ = 2700 ${\mathrm{kg}/m}^{3}$.

**Figure 3:**
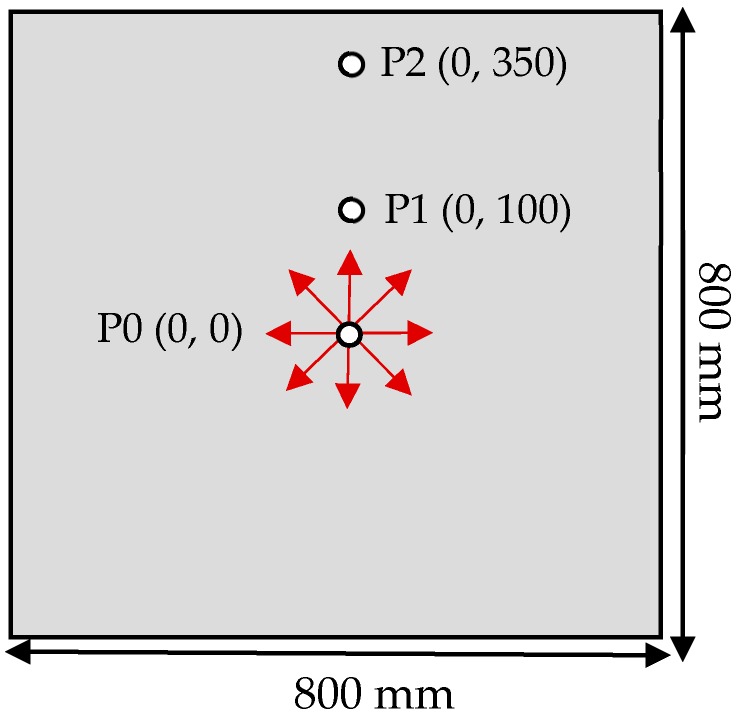
PZT transducer configuration to measure the S_0_ mode group velocity v.

Each PZT transducer had a diameter of 5.4 mm and could excite or sense the Lamb wave signals in radially outward directions. In order to accurately characterize the scattering of Lamb waves after interacting with the damage, an FE mesh having at least 8–12 nodes per Lamb wavelength was required [42]. The plate was meshed with 3D shell elements S4R with the element size of 0.5 mm in all simulation models in this work, which gave at least 28 elements per wavelength. The time step for dynamic calculation must be less than the ratio of the minimum distance of any two adjoining nodes to the maximum wave velocity [6], which, for this case, was the velocity of the S_0_ mode. Therefore, a time step of 0.02 μs was used to accurately simulate the Lamb wave propagation for a total time of 150 μs. The simulation model was first validated by calculating the S_0_ mode group velocity v in Equation (4) by using the transducer configuration shown in Figure 3. The central transducer at point P0 provided the excitation, and the transducers at P1 and P2 were used to acquire the response signals.

**Figure 4:**
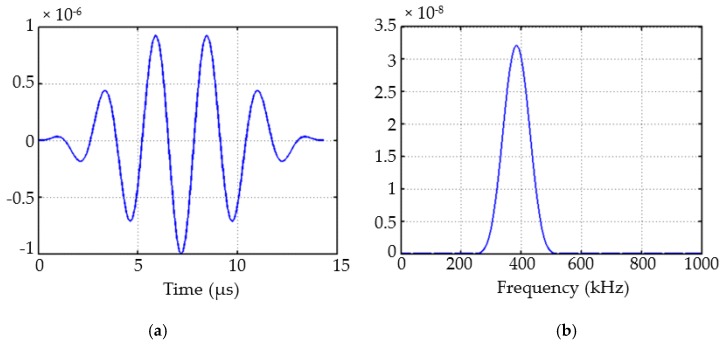
The excitation signal: (**a**) time domain and (**b**) frequency domain.

**Figure 5:**
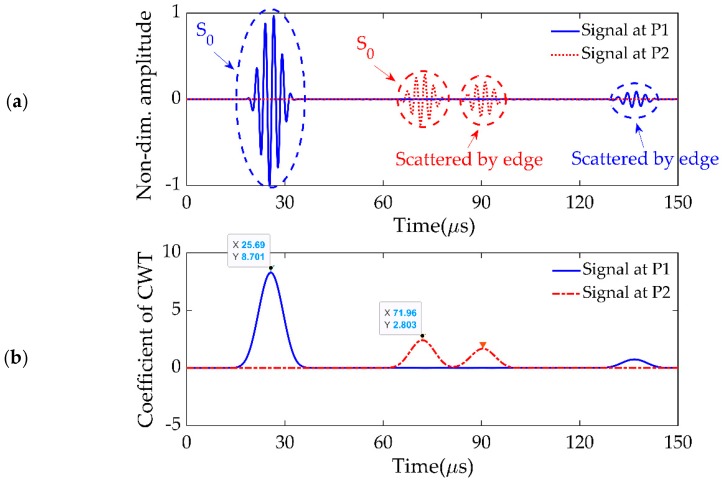
(**a**) Signals received at P1 and P2. (**b**) Continuous wavelet transformation (CWT) of signals received at P1 and P2.

The excitation signal was an in-plane force with a 5.5-cycle sinusoidal tone burst modified by a Hanning window at a central frequency of 383 kHz (as shown in Figure 4), which was generated at the center of transducer P0 to excite the Lamb waves. For the excitation frequency of 383 kHz and the plate thickness of 1.293 mm, the frequency–thickness product f.d was 514 Hz-m, shown as a red dashed line in Figure 2. This point lay in the low-frequency range of the dispersion curve, and both the S_0_ and A_0_ wave modes existed. The S_0_ mode was clearly the one traveling with higher group velocity, and it could be easily separated from the A_0_ mode at this frequency. To extract the proper wave information from the complicated wave signals, a CWT, Gabor wavelet [43], was chosen for signal processing. The signals received at P1 and P2 are plotted in Figure 5a after converting them to the nondimensional form by normalizing with the maximum amplitude, and their CWTs are presented in Figure 5b. 

The first arrival peaks were the direct excitation signal S_0_ at P1 and P2, received at the time $t_{1}=25.69\;\mu s$ and $t_{2}=71.96\;\mu s$, respectively, and the remaining two peaks were the boundary reflections. Therefore, the traveling time of Lamb waves from P1 to P2 was the difference between $t_{1}$ and $t_{2}$, namely, 46.26 μs. Because the distance between these points was 250 mm, the velocity was calculated as $5.404\;\times \;10{}^{3}m/s,$ which was almost the same as that obtained from the dispersion curve in Figure 2 (red dash line; i.e., $5.391\;\times \;10{}^{3}m/s)$. This means that the numerical model had good accuracy in calculating Lamb wave propagation, and CWT was suitable for accurately calculating the delay time which was essential for elliptical reconstruction. 

### 3.3. Detection Strategy

**Figure 6:**
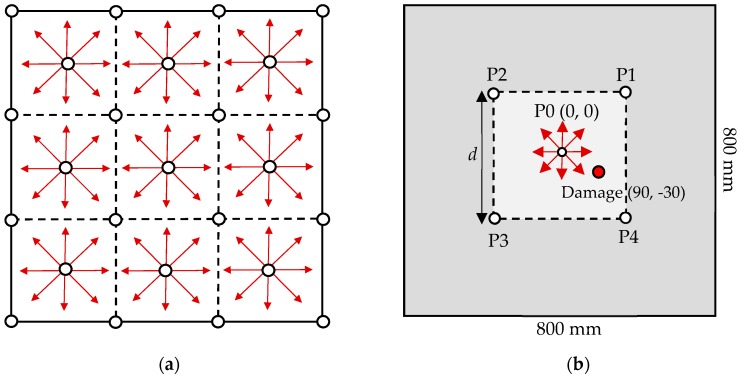
(**a**) A network of PZT transducers embedded in a large structure. (**b**) A single damaged cell.

The transducers can be arranged as a network of measuring points on a large structure, such as the wing of an aircraft or a wind turbine blade. In fact, many modern structures need to be monitored continuously during their service life, and transducers have already been embedded for in situ structural health detection [44,45]. The current method proposes a simple network of transducer detection cells where each square cell is comprised of five transducers: four at the corners of the square and one at the center, as shown in Figure 6a. At the first stage of damage detection, only the central transducer of each cell is excited to locate the damaged cell. Therefore, we only considered the single damaged cell with four PZT transducers located at points P1–P4, as shown in Figure 6b. 

**Figure 7:**
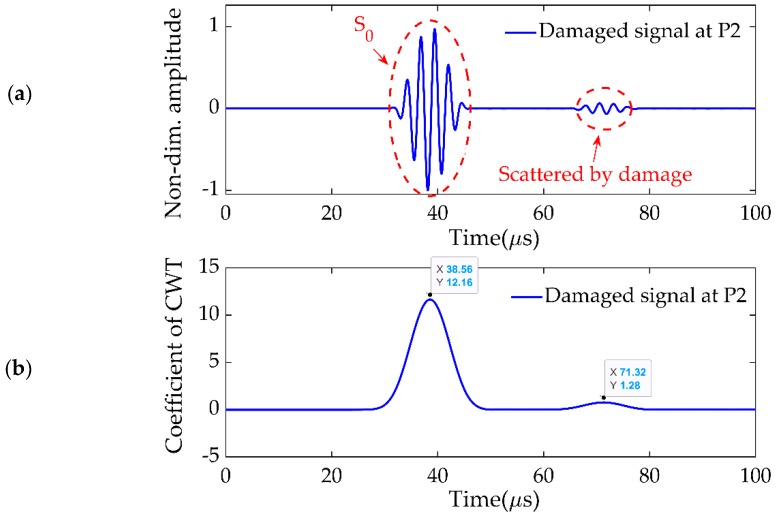
(**a**) Signal received at P2 in the damaged plate. (**b**) CWT of (**a**).

The minimum size of the estimated damage should be equal to or greater than half of the wavelength of the Lamb wave. For 383-kHz frequency excitation and $5.404\;\times \;10{}^{3}m/s$ group velocity, the wavelength was 14 mm. So, 7-mm-diameter circular damage, as a through-hole in the plate, was located at (90,−30) mm, and the distance *d* between the corner transducers was 240 mm, as shown in Figure 6b. The central transducer at P0 excited the Lamb wave signals, and the four corner transducers recorded the signals in the damaged plate. The signal received at P2 in the damaged plate is presented in Figure 7a, which included two fluctuations. The first one was the direct excitation signal S_0_, while second one was the signal scattered by the damage. The result after CWT is shown in Figure 7b, and the difference between the first and second peaks was the delay time $\Delta t_{02}=\;32.76\;\mu s$.

**Figure 8:**
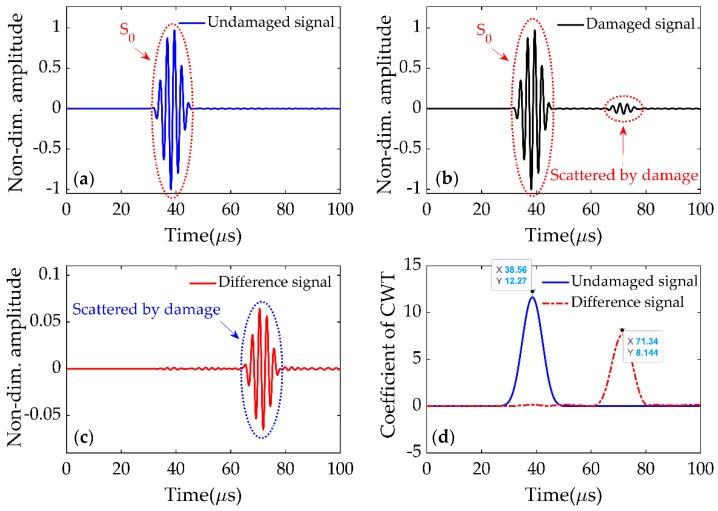
(**a**) Undamaged signal at P2. (**b**) Damaged signal at P2. (**c**) Difference between undamaged and damaged signals. (**d**) CWT of undamaged and difference signals.

In order to verify the delay times, the signal received in the damaged plate was compared with the baseline signal from the undamaged plate. The undamaged signal, shown in Figure 8a, was received at P2 in the intact plate, while the damaged signal at the same point is shown in Figure 8b. The difference between undamaged and damaged signals (shown in Figure 8c) clearly indicated the presence of damage in the plate. The CWTs of undamaged and difference signals are shown in Figure 8d, and the delay time was calculated using the first peaks of both signals. The delay time $\Delta t_{02}$ was 32.76 μs, which was same as that from Figure 7b. It indicated that the strategy adopted in Figure 7b to calculate the delay time without the baseline signal was accurate. Similarly, the delay time $\Delta t_{0i}$(i=1,…,4) could be calculated and is listed in Table 1.

**Figure 9:**
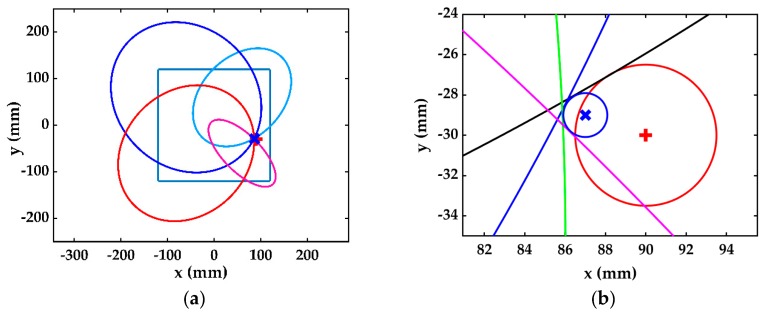
(**a**) Damage estimation in single-stage detection method. (**b**) Estimated (blue) and actual (red) damage.

The four ellipses described by Equation (4) are drawn in Figure 9a using the delay times given in Table 1, and the known values of group velocity and transducer spacing. The damage was assumed to be a circle tangent to these four ellipses. The center of this circle is the damage location, and the diameter of the circle represents its size. The blue circle in Figure 9b represents the damage estimation using this method, while the red circle is the actual damage. The damage location estimated through the single-stage damage detection method was (87,−29) mm, while the size was 2.18 mm. The relative error in damage location and size were 3.33% and 68.92%, respectively. It was proposed to excite the central transducer of each cell separately to find the damaged cell in the first stage of the damage detection. In the subsequent stages, the damage was evaluated locally inside the damaged cell. 

### 3.4. Influence of the Detection Cell Size

**Figure 10:**
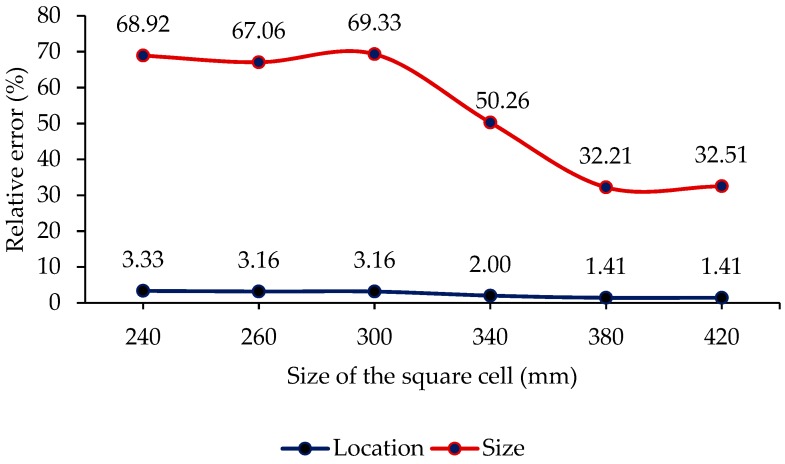
Effect of the detection cell size on the accuracy of the results.

The effect of the detection cell size on the measuring accuracy was studied by estimating the same damage, 7-mm diameter at (90,−30) mm, for different transducer spacings *d*. The spacings considered were *d* = 220, 240, 260, 300 340, 380, and 420 mm. The relative error in evaluating the damage is plotted in Figure 10. The results indicated that there was a significant improvement in the estimation of damage size as the size of the detection cell increased beyond 300 mm. The reason is that the longer distance reduced the measurement error of delay time over the spacing ∆*t/d*, as both sides of the nondimensional Equation (4) are divided by the distance *d* to calculate the damage location (*x*, *y*). The other reason may be that the S_0_ mode was better separated from the A_0_ mode beyond *d* = 300 mm and reduced the inaccuracy in calculating the delay time. There was also slight improvement in the accuracy of location estimation. A recent study has also presented a similar trend of a decrease in error as the distance from the actuator increases [46]. Therefore, the damage location and size can be better estimated using a bigger cell if the attenuation of the Lamb wave is lower.

## 4. Multistage Damage Detection Method

**Figure 11:**
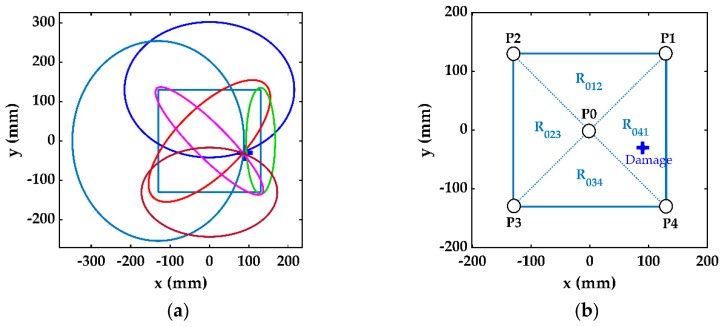
(**a**) Six additional ellipses in multistage detection. (**b**) Identification of damaged subcell.

The central transducer at P0 was excited during the single-stage damage detection, and the four corner transducers received the signals. However, each PZT transducer could, in fact, both excite and receive the Lamb wave signals. If each corner PZT transducer was also excited, and the other corner transducers were used to detect the signal, then it provided six additional delay times, listed in Table 2, which provided six additional ellipses around the damage, as shown in Figure 11a. 

To analyze the results, the detection cell can be divided into four triangular subcells formed by the transducer locations. These subcells are represented by R_ijk_ in Figure 11b, where the subscripts “ijk” are the identifiers of the three transducers forming the triangular subcell. Even though the relative error of the damage size estimation was unacceptably large in the single-stage detection, the method could accurately locate the damage location and, thus, the damaged subcell. For the simulated case, the results obtained in single-stage damage detection indicate that the damage was contained in subcell R_041_, as shown in Figure 11b. 

### 4.1. The Two-Stage Damage Detection Method

**Figure 12:**
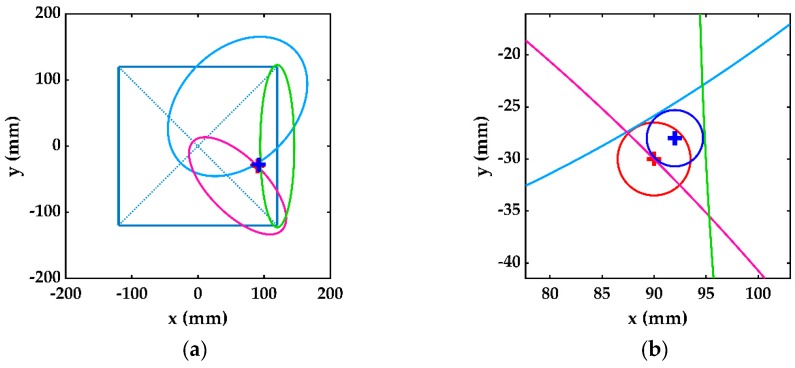
(**a**) Ellipses formed in the second-stage damage detection method. (**b**) Estimated (blue) and actual (red) damage.

The damaged cell and subcell R_041_ were located in the first stage of detection. In the second stage, the damage was estimated through three ellipses formed in the damaged subcell to improve the size estimation. These ellipses were formed using the delay time values $\Delta t_{01},\;\Delta t_{04},\;\mathrm{and}\;\Delta t_{14}$ between the points P0–P1, P0–P4, and P1–P4, respectively. The first two ellipses were provided by the single-stage detection method, while the additional third ellipse was formed between the corner points P1 and P4 using the delay time $\Delta t_{14}$. The delay time $\Delta t_{14}$ was calculated by exciting any of the corner transducers (P1 or P4) and sensing the signal at the other corner one. The ellipses used for the second-stage are shown in Figure 12a. The location and size of the damage in the second-stage were calculated as (92,−28) mm and 5.41 mm, respectively. The estimated (blue) and actual (red) damage is shown in Figure 12b, with relative errors in damage location and size of, respectively, 2.98% and 22.67%. It is evident that in the second-stage, both the damage location and size were estimated more accurately than in the single-stage detection method.

### 4.2. The Three-Stage Damage Detection Method

**Figure 13:**
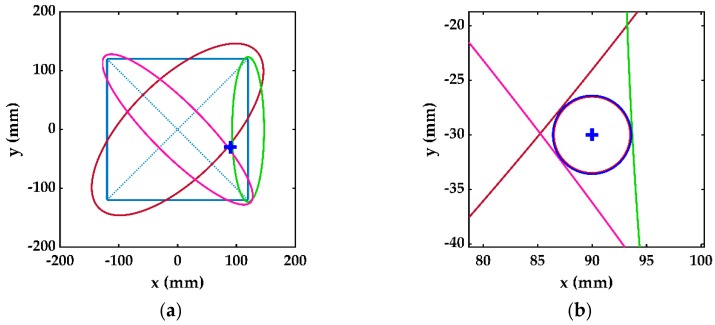
(**a**) Three corner ellipses in the third-stage damage detection. (**b**) Estimated (blue) and actual (red) damage.

The single-stage damage detection method located the damaged cell and subcell, while the damage location and size estimation were improved in the second-stage by one additional corner ellipse. In order to further improve the damage size estimation in the third-stage of damage detection, besides the additional ellipse in the second stage, two more ellipses were constructed between the corresponding corner transducers of subcell R_041_. These ellipses were formed using the delay times $\Delta t_{13}\;\mathrm{and}\;\Delta t_{24}$ between the transducers, respectively, at points P1–P3 and P2–P4. These three ellipses between the corner transducers of subcell R_041_ are shown in Figure 13a and were used to calculate the damage location and size of, respectively, (90,−30) mm and 7.19 mm. The actual and estimated damage in the third-stage damage detection are shown in Figure 13b, indicating almost vanishing errors, respectively, of 0% and 2.66% for damage location and size.

## 5. Application for Multiple Damage Detection

In the case of multiple damage in a plate-like structure, the arrangement of transducers in the form of a network of cells, as proposed in Figure 6a, can help to estimate each damage in its cell. For the case when multiple damages exist in one cell, the further division of a cell into four triangular subcells will probably separate them, as the size of the subcell is very small compared with the whole structure. However, in an extreme case, there can be multiple damages so close to each other that they may exist in one subcell.

**Figure 14:**
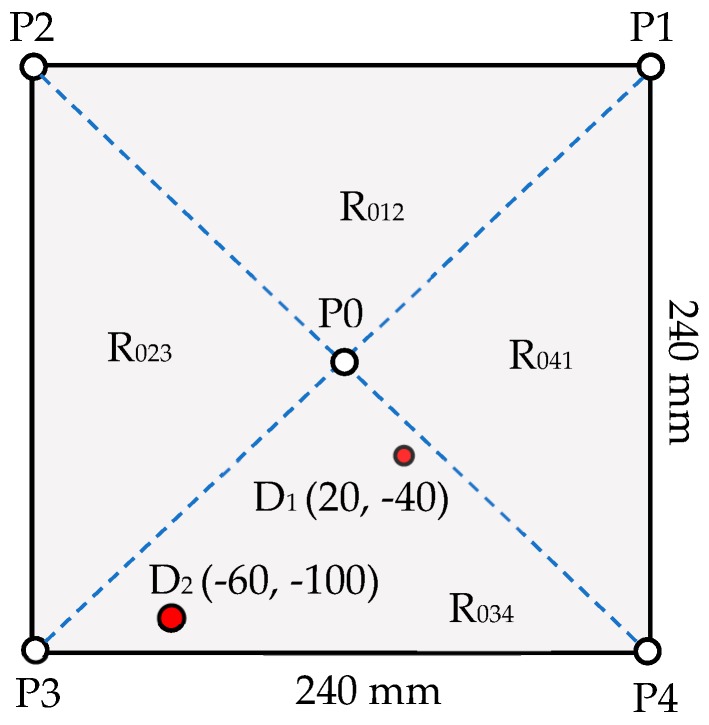
A cell with multiple damages of different sizes in one subcell.

**Figure 15:**
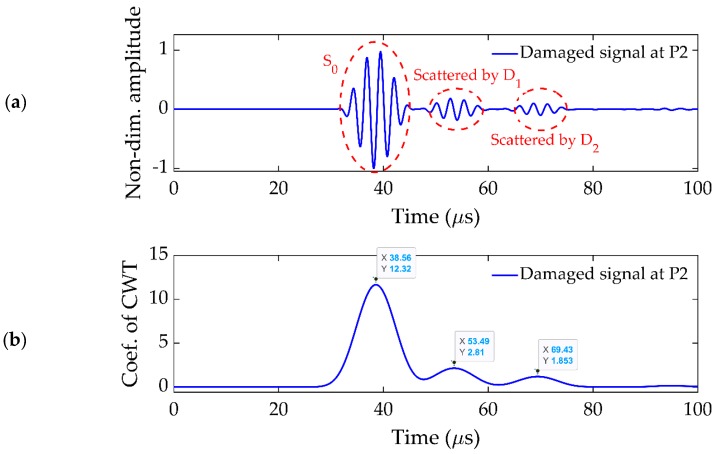
(**a**) Signal received at P2 in plate with multiple damages. (**b**) CWT of (**a**).

For such a case, there will be as many damage-scattered peaks in Figure 7 as the number of damages in the plate, and each peak will provide the delay time caused by the corresponding damage. Therefore, based on the delay times calculated from the damage-scattered wave signals, all the ellipses would converge around their corresponding damage. An example multiple damage existing in one subcell is presented in Figure 14, where two damages, D_1_ and D_2_, of 10 and 8 mm diameters, existed at (20,−40) and (−60,−100) mm, respectively. The signal received at P2 when the central transducer at P0 was excited in the first stage of detection is presented in Figure 15a, which shows two damage-scattered peaks. The CWT of this signal is presented in Figure 15b, which helped to calculate the delay time for each damage using the strategy discussed in Section 3.2.

**Figure 16:**
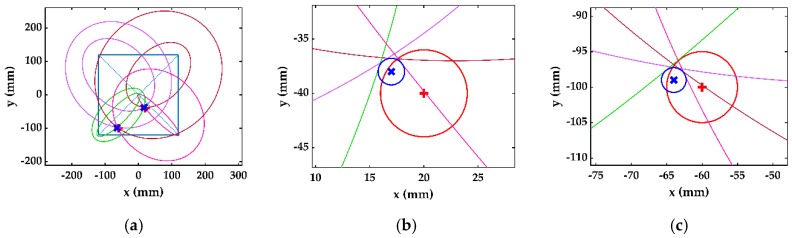

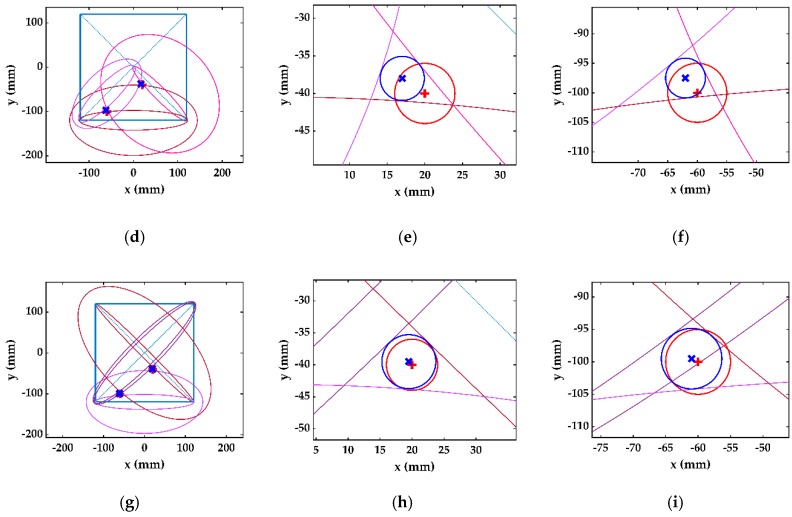
Multiple damage detection, estimated (blue) and actual (red): (**a**) single-stage detection; (**b**) D_1_ at first stage; (**c**) D_2_ at first stage; (**d**) two-stage detection; (**e**) D_1_ at second stage; (**f**) D_2_ at second stage; (**g**) three-stage detection; (**h**) D_1_ at third stage; (**i**) D_2_ at third stage.

Similarly, there were two delay time values for each actuator–receiver pair, which formed a total of eight ellipses in the first stage using four actuator–receiver pairs. Each ellipse from each pair converged about one damage, which made four ellipses converge about D_1_ and the other four about D_2_. It is not important to know that which delay time or ellipse belongs to which damage; the designed algorithm estimates each damage using the four ellipses converging at one location. Figure 16a shows the multiple damage detection in the single stage, and the comparison of estimated and actual damage is shown in Figure 16b for D_1_ and Figure 16c for D_2_. Figure 16d shows the ellipses formed in second stage, and the comparison of estimated and actual damage for D_1_ and D_2_ is given in Figure 16e,f, respectively. Similarly, Figure 16g shows the ellipses formed in the third stage, and the comparison of estimated and actual damage for D_1_ and D_2_ is in Figure 16h,i, respectively.

The relative error in the location and size estimation for each damage is presented in Table 3. The results indicate that there was improvement in damage estimation in the second and third stages of the detection for multiple damages in one subcell. However, the relative errors in the case of multiple damages were slightly higher than the single damage case.

## 6. Experimental Evaluation

**Figure 17:**
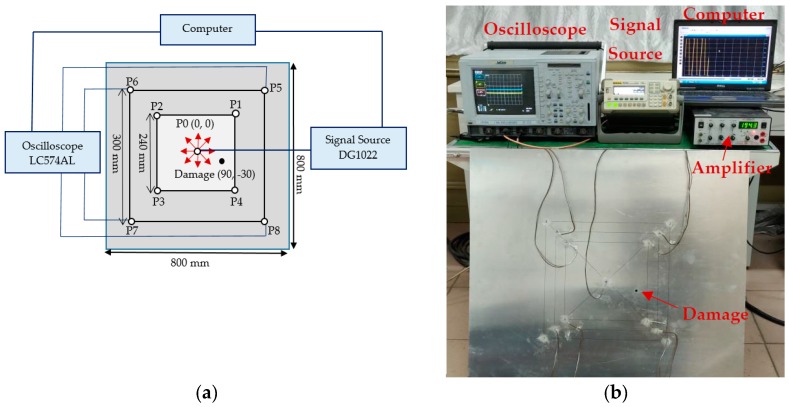
Experimental setup: (**a**) schematic diagram and (**b**) laboratory diagram.

The accuracy of the proposed multistage damage detection method was further verified through experimental investigation. The plate dimensions, material properties, and transducer configuration were exactly the same as those in the numerical simulation shown in Figure 6b. The damage was a punched hole of 7-mm diameter at the location of (90,−30) mm. The PZT wafers used in the experiments were made of PSN-33 with a density of 7.70 × 10^3^ kg/m^3^ and a diameter of 5.4 mm. The PZT wafers had a resonance frequency of 383 kHz and had the ability to excite/sense the Lamb wave signals in radially outward directions. The experiments were performed for two PZT configurations with *d* = 240 mm and *d* = 300 mm, respectively. The schematic diagram of the experimental setup for multistage damage detection is shown in Figure 17a, and the real-time experimental setup is shown in Figure 17b. A signal generator (RIGOL, DG1022) was programmed to generate the signal shown in Figure 4a at the excitation transducer P0, and the signal was amplified to 45 V using a power amplifier (KROHN-HITE, 7602M). A four-channel oscilloscope (LeCroy, LC574AL) was used to record the response of each transducer. The oscilloscope captured the response signals over a sampling time of 200 μs at a sampling rate of 1 GHz. Every signal was acquired for an average of 500 times to reduce the noise.

During the single-stage of damage detection, the central transducer at point P0 was excited to induce the Lamb wave, and transducers P1–P4 (*d* = 240 mm) and P5–P8 (*d* = 300 mm) were used to measure the received signals. In multistage damage detection, the corner PZT transducers at P1, P2, P5, and P6 were also used to excite Lamb waves for the second and third stages of damage detection.

**Figure 18:**
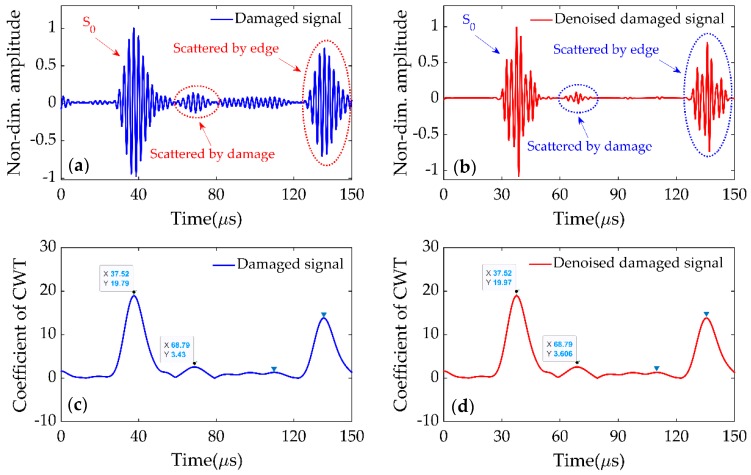
(**a**) Received signal at P2. (**b**) Denoised signal of (**a**). (**c**) CWT of signal (**a**). (**d**) CWT of signal (**b**).

The signal received at P2 was processed in dimensionless form and is shown in Figure 18a, which showed fluctuations caused by the scattering from the damage and the plate’s edge. The acquired signal shown in Figure 18a was then processed by Sym8 wavelet function in *MATLAB* to reduce the noise. The denoised signal is presented in Figure 18b, which looks similar to the signal received at the same point during the simulation (as shown in Figure 7b). The CWTs of recorded and denoised signals are presented in Figure 18c,d, respectively, and the delay time was calculated as $\Delta t_{02}=\;31.27\;\mu s,$ which was close to the delay time $\Delta t_{02}=\;32.76\;\mu s$ calculated through FE simulations.

The delay times, similarly calculated through experiments for signals received at all transducers, are shown in Table 4.

The relative error in damage estimation at different stages of detection through simulations and experiments are compared with the actual location and size of the damage in Table 5.

The experimental results of damage location and size for single-stage damage detection in the smaller detection cell had relative errors, respectively, of 15.28% and 128.56%. The relative errors in the same cell were reduced in the second stage to, respectively, 5.13% and 56.64%, and in the third stage to, respectively, 3.16% and 24.20%. A similar trend in the improvement of damage detection was found in the larger detection cell. The damage assessment accuracy of the multistage method was significantly higher than the single-stage detection in both cases; in particular, there was a significant improvement in the damage size detection.

## 7. Discussion of Results

During the numerical simulation, the excitation of the central PZT transducer in the single-stage damage detection method caused Lamb waves to propagate radially outward in all directions, and be scattered by any damage in the plate. The single-stage method efficiently located the damage (3.33% of error for *d* = 240 mm). The relative error in damage size estimation in the single-stage method was unacceptable (68.92% for *d* = 240 mm). However, the accuracy of damage location can help to identify the damaged cell, and the triangular subcell containing the damage. So, the single-stage damage detection method can directly obtain the size and location information of the damage, and it can locate the damaged subcell for the subsequent detection stages, especially for improving the estimation accuracy of damage size.

Based on the same transducer configuration, the second stage of damage detection could be conducted to improve the damage estimation by two ellipses from the single-stage detection, and one additional corner ellipse around the triangular subcell. The additional ellipse was obtained by exciting a corner transducer, and receiving at the other corner transducer separately in this subcell. With the three ellipses, the second stage of damage detection could reduce the relative errors in damage location and size, respectively, from 3.33% to 2.98% and from 68.92% to 22.67%.

Similarly, using the same transducer configuration, the third stage of damage detection was carried out by using the additional ellipse from the second stage and two extra ellipses, which were obtained by exciting the two corner transducers of the damage subcell and receiving at the other two corner transducers in the diagonally opposite direction. Using these three ellipses could reduce the relative errors from the second to the third stage in damage location and size, respectively, from 2.98% to 0% and 22.67% to 2.66%. 

From the trend shown in Figure 10, it can be concluded that the bigger ellipses obtained by larger detection cells could provide a better estimation of damage. Therefore, compared with the fast detection of the single-stage method with only a one-time excitation, the second stage could obtain better results by including one additional large ellipse from one extra excitation, and the third stage had the best damage estimation from two additional large ellipses by two extra excitations. This is because the single-stage method obtained the ellipses with transducer spacing $\frac{\sqrt{2}}{2}d$, the additional ellipse in the second stage was obtained by the corner transducers with large spacing *d*, and the third stage included two extra ellipses with larger transducer spacing $\sqrt{2}d.$ It can be noticed that the bigger ellipse touching the damage, as shown in Figure 13b, was almost a straight line and more accurately defined the tangent to the damage periphery than the smaller ellipse, as shown in Figure 12b. As a result, depending on the location of the damage, the accuracy of the damage size was limited in the single-stage damage detection method by the size of the detection cell. In addition, the longer distance reduced the error in calculating the delay time by Equation (4). However, this was restricted by the attenuation of Lamb wave propagation. 

The same conclusion can be confirmed by the experimental results in Table 5; that is, the bigger ellipses formed in the large detection cell (*d* = 300 mm) could obtain better results than the small ones (*d* = 240 mm) at the same stage, and the detection accuracy could be improved at the higher stage in each detection cell. Even if the three-stage detection could provide accurate damage information, the excitation of the central transducer in single-stage damage detection was still important to locate the damaged cell and subcell used in the second and third damage detection stages. Actually, the utilization of third-stage detection depends on the nature of the problem and the accuracy requirements in practice.

Comparing Figure 18c,d, the difference is hard to identify. This means the CWT had a better signal-to-noise ratio, and could extract the time-frequency feature exactly from the noisy signal. This is also the reason why CWT could process the Lamb wave signals of the damaged plate directly without a baseline. Generally, a greater value of error is expected in the larger cell due to the dispersion of Lamb waves, which leads to the inaccuracy in determining the delay time. However, the accuracy of this damage detection method improves as the size of the detection cell increases. This is because the CWT is a better time-frequency representative and can depress the dispersive characteristics effectively. 

## 8. Conclusions

The Lamb-wave-based multistage damage detection method introduced in this research can accurately quantify both the location and size of damage. According to the PZT transducer network in practice, each square transducer cell can be used to identify the damage locally. The single stage can be performed at each cell simultaneously in a large PZT network to estimate the damage location and size and, further, to identify the damaged cell and subcell. In order to further improve the damage size detection, the second stage can be carried out only in the damaged subcell. If further improvement in size estimation is required, the third stage can be utilized for higher accuracy achievement. Compared with existing ellipse-based methods, the amount of data collected in this method is very small due to a small number of transducers used to form the damaged cell. Four excitations are required from the single to the third stage: one excitation each in the single and second stages, and two excitations in the third stage of detection. The data collected in one stage is also reused in subsequent stages without any other additional tools or techniques. Therefore, the proposed method can save both computational time and evaluation cost during the NDT/SHM process. Further, the proposed method does not require a baseline signal or any additional transducers in higher stages of detection, which makes it suitable for on-line monitoring. The transducer configuration, signal-processing, and outer tangent circle method introduced in the single-stage damage detection method remain exactly the same in the second and third stages of damage detection. The method is relatively simple and straightforward to implement for detection of circle-like damage in thin plate structures used in civil, automobile, aerospace, ship building, and wind turbine industries.

## Figures and Tables

**Table 1:** Delay time for transducers at P1–P4 with P0 excitation.

	Δ*t*_01_	Δ*t*_02_	Δ*t*_03_	Δ*t*_04_
**Delay time (μs)**	13.25	32.76	27.30	3.91

**Table 2:** Delay time during multistage detection.

	Δ*t*_12_	Δ*t*_13_	Δ*t*_14_	Δ*t*_23_	Δ*t*_24_	Δ*t*_34_
**Delay time (μs)**	38.26	7.18	1.07	44.68	2.12	25.89

**Table 3:** Comparison of results for multiple damages.

Detection Method	Error in Location Estimation (%)		Error in Size Estimation (%)	
	D_1_	D_2_	D_1_	D_2_
**Single-stage**	8.06	3.54	69.41	65.19
**Two-stage**	8.06	2.74	26.75	32.6
**Three-stage**	1.58	0.96	5.31	6.68

**Table 4:** Delay times (µs) calculated through experiments.

Cell Size	Delay Times (µs)						
*d* = 240 mm	**Δ*t*_01_**	**Δ*t*_02_**	**Δ*t*_03_**	**Δ*t*_04_**	**Δ*t*_13_**	**Δ*t*_14_**	**Δ*t*_24_**
	13.80	31.27	30.31	2.93	6.89	1.27	2.51
*d* = 300 mm	**Δ*t*_05_**	**Δ*t*_06_**	**Δ*t*_07_**	**Δ*t*_08_**	**Δ*t*_57_**	**Δ*t*_58_**	**Δ*t*_68_**
	12.68	33.41	29.36	3.12	6.72	1.83	2.47

**Table 5:** Comparison of simulation and experimental results with the actual damage location and size.

Detection Size *d* (mm)	Detection Method	Error in Experimental Results (%)		Error in Numerical Results (%)	
		Location	Size	Location	Size
240	Single-stage	15.28	128.56	3.33	68.92
	Two-stage	5.13	56.64	2.98	22.67
	Three-stage	3.16	24.20	0	2.66
300	Single-stage	12.58	107.3	3.16	69.33
	Two-stage	5.17	43.41	2.87	17.53
	Three-stage	2.94	18.66	0	2.34
